# Unicornuate Uterus with Noncommunicating Cavitary Horn

**DOI:** 10.5334/jbr-btr.1160

**Published:** 2016-09-26

**Authors:** Mathieu Lefere, Sofie De Vuysere, Yves De Bruecker, Annick Demeyere

**Affiliations:** 1 Imeldaziekenhuis Bonheiden, BE

**Keywords:** unicornuate uterus, müllerian duct anomaly, endometrioma, MRI, haemoperitoneum

## Observation

A 51-year-old nulliparous woman was referred to our department for an MRI scan of the pelvis in the work-up of pathologically proven cervical cancer. A HPV (humane papilloma virus) DNA test was positive for high-risk HPV types. Pathological analysis of a cervical biopsy showed poorly differentiated squamous cell carcinoma. The patient had a personal history of left renal agenesis, a presumed Müllerian duct anomaly, and surgery for endometriosis. At our department, the routine scanning protocol for cervical cancer staging consists of sagittal, para-axial, and paracoronal T2 HASTE images adjusted to the cervical axis and axial diffusion-weighted images. Based on this MRI exam, the tumor was locally staged as cT2bN1. Treatment consisted of surgical removal of a large external iliac adenopathy followed by concomitant radio-chemotherapy.

**Figure 1:**
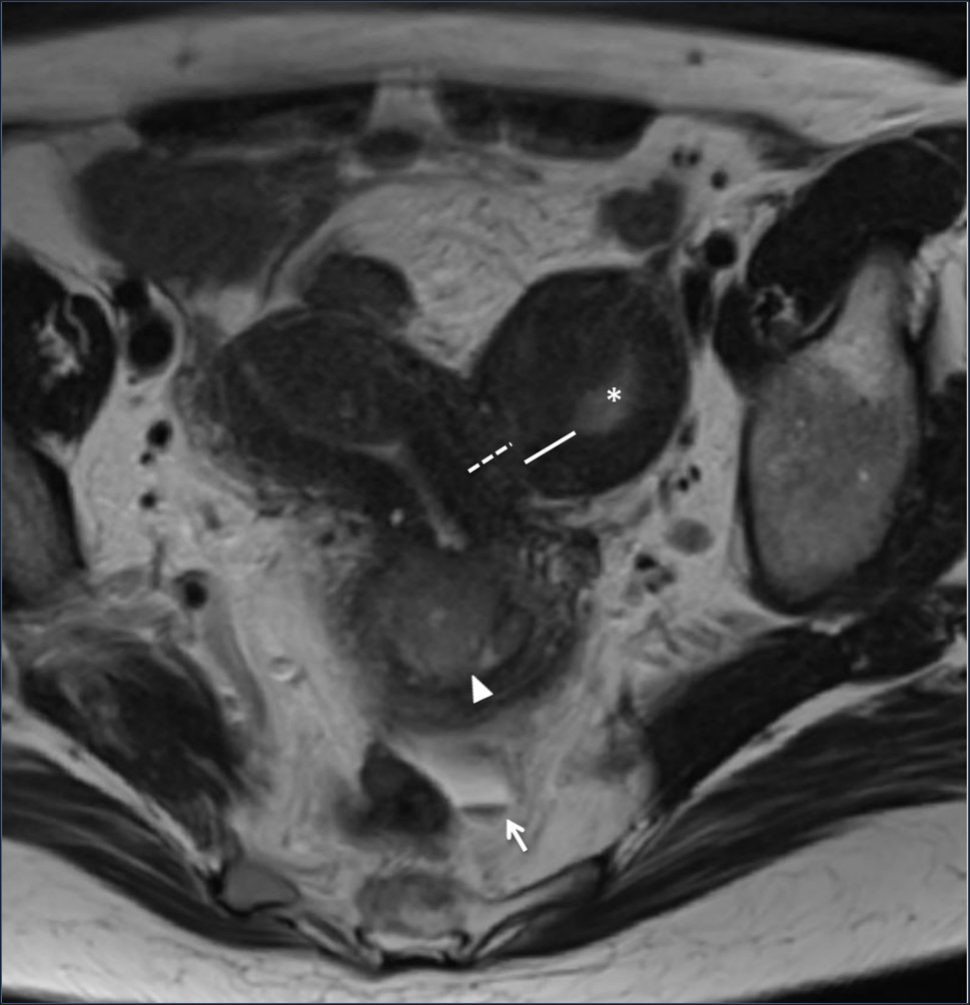


**Figure 2:**
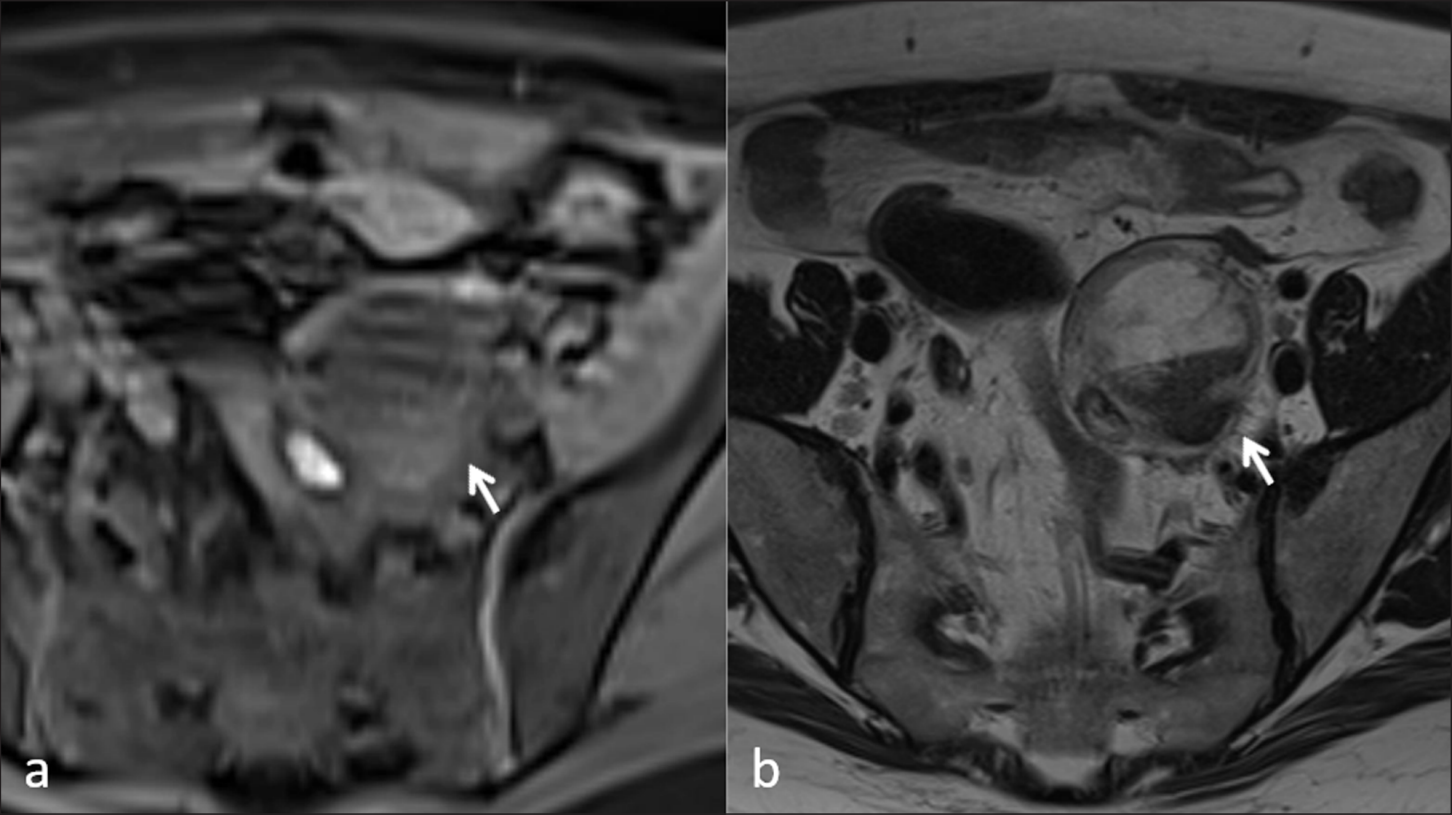


In this patient, MRI also confirmed the presence of a uterine anomaly (Figure 1). The left uterine horn contained a distinct cavity (*) and junctional zone (line) that were separated from the right horn and corpus by a layer of myometrial tissue (white dashed line). In the right uterine horn, the junctional zone was focally thickened. A small amount of fluid with a T2-hypointense component was also seen in the recto-uterine pouch (arrow). Additionally, a complex thick-walled cystic mass was found in the left iliac fossa, adjacent to the left uterine horn. To further characterize this unknown lesion, axial T1 images with fat saturation were made (Figure 2). A distinct T1 hyperintense and T2 hypointense layer was seen within this mass (arrows).

Based on these observations, the diagnosis of right unicornuate uterus with noncommunicating left cavitary horn was made. The junctional zone thickening was compatible with adenomyosis. The complex cystic mass was consistent with endometrioma. A small amount of hemoperitoneum was the final important secondary finding.

## Comment

The female reproductive organs develop during the sixth week of gestation, when the paired Müllerian (or paramesonephric) ducts fuse to create the uterus, cervix, and upper two-thirds of the vagina. Unicornuate uterus is a result of abnormal or failed development of one of the Müllerian ducts. Unilateral renal agenesis is the most frequently associated urinary tract abnormality [1].

Four subtypes of unicornuate uterus have been described, based on the presence or absence of a rudimentary uterine horn, which may or may not communicate with the normal horn. If present, functional endometrial tissue within a rudimentary horn puts the patient at higher risk for endometriosis, hematometra, and hematosalpinx, as well as adenomyosis. Fetal implantation can occur in a noncommunicating rudimentary horn, but it will generally result in a life-threatening uterine rupture. Therefore, the correct diagnosis of this entity has important clinical implications, especially in young patients with a desire for pregnancy.

MRI, with its excellent soft tissue contrast and complete lack of radiation exposure, allows accurate diagnosis of all subtypes of unicornuate uterus. Unicornuate uterus with cavitary noncommunicating horn can be classified as a Müllerian duct anomaly type A1b, according to the American Fertility Society. Differential diagnosis includes adnexal mass or pedunculated uterine fibroma.
